# ﻿Phylogenetic analysis shows that *Pyrenula* (Pyrenulaceae) diversity is larger than expected: three new species and one new record discovered in China

**DOI:** 10.3897/mycokeys.110.131741

**Published:** 2024-11-13

**Authors:** Mingzhu Dou, Jiechen Li, Yongshun Hu, André Aptroot, Zefeng Jia

**Affiliations:** 1 College of Agriculture and Life Sciences, Liaocheng University, Liaocheng 252059, China Liaocheng University Liaocheng China; 2 Laboratório de Botânica, Liquenologia, Instituto de Biociências, Universidade Federal de Mato Grosso do Sul, Avenida Costa e Silva s/n, Bairro Universitário, CEP 79070-900, Campo Grande, Mato Grosso do Sul, Brazil Universidade Federal de Mato Grosso do Sul Campo Grande Brazil

**Keywords:** Chemical substances, diversity, morphology, new taxa, phylogeny

## Abstract

The lichenised fungal genus *Pyrenula* is a very common crustose lichen element in tropical to subtropical forests, but little research has been done on this genus in China. We carried out an integrative taxonomic study on *Pyrenula* in China using morphological, anatomical, chemical characters, and molecular data (ITS, nuLSU, mtSSU). Three new species with muriform ascospores containing red oil when over-mature were found: *Pyrenulasubmacularis***sp. nov.**, *P.yunguiensis***sp. nov.** and *P.rufotetraspora***sp. nov.** Molecular data and TLC results of *P.macularis* and *P.breutelii* are for the first time reported and show that they are not synonyms. This is the first report of *P.breutelii* in China. Contrary to the previous reports of this genus, we found lichen substances in all the five species in this study, seemingly terpenoids. A key for the *Pyrenula* species reported in China is provided.

## ﻿Introduction

The lichen genus *Pyrenula* Ach. (Pyrenulaceae) was first established by Acharius, with *Pyrenulanitida* (Weigel) Ach. as the type species (Acharius 1814). *Pyrenula* grows on bark mainly in tropical and subtropical forests (Aptroot 2012; Mendonça et al. 2016). The genus is characterised by perithecioid ascomata, with or without pseudocyphellae, with or without lichexanthone or anthraquinones, with distoseptate, transversely septate or (sub)muriform ascospores. UV-reaction of thallus, presence of hamathecium inspersion and the shape of the ascospore lumina (especially whether there is an endospore between the end lumina and the wall) are also important characters (Aptroot 2012; Mendonça et al. 2016).

Based on integrative taxonomic studies applying molecular, morphological, anatomical, and chemical characters, including the long-overlooked characters inspersion and iodine reaction of the hamathecium, we reported three new species of *Pyrenula* (*P.inspersa*, *P.thailandicoides* and *P.apiculata*), which have 3-septate ascospores with red or orange oil when over-mature (Dou et al. 2024). The presence of red or orange oily granules, which occur in over-mature ascospores of some *Pyrenula* species, was first recognised by Harris (Harris 1989). He pointed out the significance of the degradation stage of spores for the taxonomy of *Pyrenula*. Aptroot et al. described the degradation process in detail: in a few species, the old spores assume a reddish tinge, the wall becomes red-brown and the remains of the lumina develop into red or orange granules (Aptroot et al. 2013). Now, a total of eleven species with red or orange oil in over-mature ascospores are known, of which seven have transversely distoseptate ascospores, viz. *P.concastroma* R.C. Harris, *P.bahiana* Malme, *P.sexlocularis* (Nyl.) Müll. Arg., *P.thailandica* Aptroot, *P.inspersa* M.Z. Dou & Z.F. Jia, *P.thailandicoides* M.Z. Dou & Z.F. Jia and *P.apiculata* M.Z. Dou & Z.F. Jia; four have (sub)muriform ascospores, viz. *P.endocrocea* Aptroot, *P.seminuda* (Müll. Arg.) Sipman & Aptroot, *P.breutelii* (Müll. Arg.) Aptroot and *P.macularis* (Zahlbr.) R.C. Harris.

In the world key of *Pyrenula* species, Aptroot accepted 169 species out of the ca. 745 named taxa in the genus (Aptroot 2012). Since then, many new species of *Pyrenula* have been described and the genus now comprises ca. 245 species (Aptroot 2012, 2021; Aptroot et al. 2012, 2013, 2018; Mendonça et al. 2016; Ingle et al. 2018; Miranda-González et al. 2022; Mishra et al. 2022; Soto-Medina et al. 2023; Lücking et al. 2023; Sipman, 2023; Dou et al. 2024), of which 46 species have so far been found in China (Aptroot and Seaward 1999; Aptroot 2003; Fu et al. 2018, 2019; Wang et al. 2018; Wei 2020; Xie et al. 2021; Dou et al. 2024; Li et al. 2024).

Here, we add three new species of *Pyrenula* with muriform ascospores with red oil when over-mature. In addition, we found that *P.macularis* is not synonymous with *P.breutelii* (Aptroot 2012). These classification results are strongly supported by molecular phylogeny. Few species of *Pyrenula* have been established based on phylogenetic result previously.

## ﻿Materials and methods

### ﻿Morphological and chemical analyses

The specimens were collected in the provinces Hunan, Fujian, Guizhou and Guangdong of China and are preserved in the Fungarium of the College of Life Sciences, Liaocheng University, China (LCUF). Morphological characters of thalli and apothecia were examined in the usual way and photographed under an Olympus SZX16 dissecting microscope with an Axio Imager. The anatomical characters were observed and measured under an Olympus BX53 compound microscope with an Olympus DP74 Imager. The lichen secondary metabolites were studied by thin-layer chromatography using solvent C (Orange et al. 2010).

### ﻿DNA extraction, PCR sequencing and phylogenetic analysis

The genomic DNA of ascomata was extracted using the Hi-DNA-secure Plant Kit (Tiangen, Beijing, China) according to the manufacturer’s protocol. The mtSSU, ITS and nuLSU regions were amplified using the primer pair mrSSU1/3R (Zoller et al. 1999), ITS1F/ITS4 (White et al. 1990; Gardes and Bruns 1993) and AL2R/LR6 (Vilgalys and Hester 1990; Mangold et al. 2008). PCR reactions were carried out in 25 µL reaction system containing 1 µL each primer solution (10 µM), 0.5 µL genomic DNA, 10 µL ddH2O, and 12.5 µL 2×Taq PCR MasterMix®. Thermocycling conditions for mtSSU comprised initial denaturation at 94 °C (3 min); 35 denaturation cycles at 94 °C (30 s), annealing at 52 °C (30 s), extension at 72 °C (1.5 min), and a final extension at 72 °C for 10 min. The PCR amplification progress for nuLSU followed Dou et al. (Dou et al. 2018). Thermocycling conditions for ITS comprised initial denaturation at 94 °C (3 min); 35 denaturation cycles at 94 °C (30 s), annealing at 52 °C (30 s), extension at 72 °C (1.5 min), and a final extension at 72 °C for 10 min. The target products of PCR were affirmed by electrophoresis on 1% agarose gels and sequenced by TsingkeBiotechnology Co.,Ltd. (Tsingtao). The newly-generated sequences were submitted to GenBank (https://www.ncbi.nlm.nih.gov/, accessed on 31 December 2025; Table 1).

**Table 1.** Information for the sequences used in this study. Newly generated sequences are shown in bold.

Species name	Specimen No.	Locality	GenBank accession number		
			ITS	nuLSU	mtSSU
*P.submacularis* M.Z. Dou & Z.F. Jia	FJ211750	China Fujian	**PP692372**	**PP692480**	—
*P.submacularis* M.Z. Dou & Z.F. Jia	FJ220211	China Fujian	**PP692377**	**PP692481**	—
*P.yunguiensis* M.Z. Dou & Z.F. Jia	GZ18096	China Guizhou	**PP692374**	**PP692478**	—
*P.yunguiensis* M.Z. Dou & Z.F. Jia	GZ18128	China Guizhou	**PP692373**	**PP692479**	—
*P.yunguiensis* M.Z. Dou & Z.F. Jia	YN221461	China Yunnan	**PP692378**	**PP692477**	—
*P.rufotetraspora* M.Z. Dou & Z.F. Jia	GZ18377	China Guizhou	**PP692371**	**PP692474**	—
*P.macularis* (Zahlbr.) R.C. Harris	HNX18016	China Hunan	**PP692368**	—	—
*P.macularis* (Zahlbr.) R.C. Harris	HNX18017	China Hunan	**PP692369**	—	—
*P.macularis* (Zahlbr.) R.C. Harris	HNX18018	China Hunan	**PP692370**	**PP692473**	**PP659691**
*P.breutelii* (Müll. Arg.) Aptroot	GD19285	China Guangdong	**PP692375**	**PP692475**	—
*P.breutelii* (Müll. Arg.) Aptroot	GD19286	China Guangdong	**PP692376**	**PP692476**	**PP659692**

The sequences of mtSSU, ITS and nuLSU were combined, and the alignment included 118 ITS sequences, 94 LSU sequences and 76 SSU sequences, representing 127 taxa. The sequences of 5 taxa were newly generated (Table 1) and the sequences of 122 taxa were downloaded in GenBank (Suppl. material 1) (Lutzoni et al. 2001; Geiser et al. 2006; Weerakoon et al. 2012; Gueidan et al. 2016). *Endocarponpusillum* and *Cyphellophoraeuropaea* were chosen as outgroup, based on previous studies (Gueidan et al. 2016). All *Pyrenula* taxa that could be found in GenBank were included in our data matrix.

The alignment of sequences for each marker (mtSSU, ITS and nuLSU) was undertaken independently by applying MAFFT 7 (Katoh and Standley 2013). We used the “maskSegment” function in the R package AlignmentFilter (Zhang et al. 2023) to mask ambiguously-aligned or overly-divergent segments (stringency-controlling parameter prob set to 0.05) and then used the “degap” function to remove sites with more than 50% gaps. The congruence of the two datasets was tested using a 70% reciprocal bootstrap criterion (Mason-Gamer and Kellogg 1996): the three matrices (mtSSU, ITS and nuLSU) were analysed separately with RaxML v.8.2.12 (Stamatakis 2014) using 100 bootstrap pseudoreplicates and implementing a GTRGAMMA model on the CIPRES Web Portal (http://www.phylo.org). The resulting trees were compared and any hard conflicts detected were eliminated by pruning sequences or taxa out of the datasets. The three single-locus alignments were concatenated in PhyloSuite v.1.2.2 (Zhang et al. 2020). The concatenated data matrix comprised 2440 characters (726 for mtSSU, 730 for ITS and 984 fornuLSU). For BI (Bayesian Inference) analysis, PartitionFinder 2 (Lanfear et al. 2017) was used to determine the best-fit model for each partition. The dataset was partitioned into gene groups, with the GTR+I+G, SYM+I+G and GTR+I+G substitution models applied to mtSSU gene, ITS gene and nuLSU gene, respectively. BI analysis was performed with MrBayes 3.2.7 (Ronquist et al. 2012). Two runs of four chains were carried out for 10,000,000 generations and trees were sampled every 1000 generations. The first 25% of the convergence runs were discarded as burn-in. Construction of the ML (Maximum Likelihood) tree was undertaken by applying RAxML v.8.2.12 (Stamatakis 2014), using 100 bootstrap pseudoreplicates and a GTRGAMMA model on the CIPRES Web Portal (http://www.phylo.org). ML bootstrap values (BS) ≥ 70% and Bayesian posterior probabilities (PP) ≥ 0.95 were considered as significantly supported. The alignments were deposited in TreeBase (http://purl.org/phylo/treebase/phylows/study/TB2:S31341).

## ﻿Results

### ﻿Phylogenetic analyses

**Figure 1.**
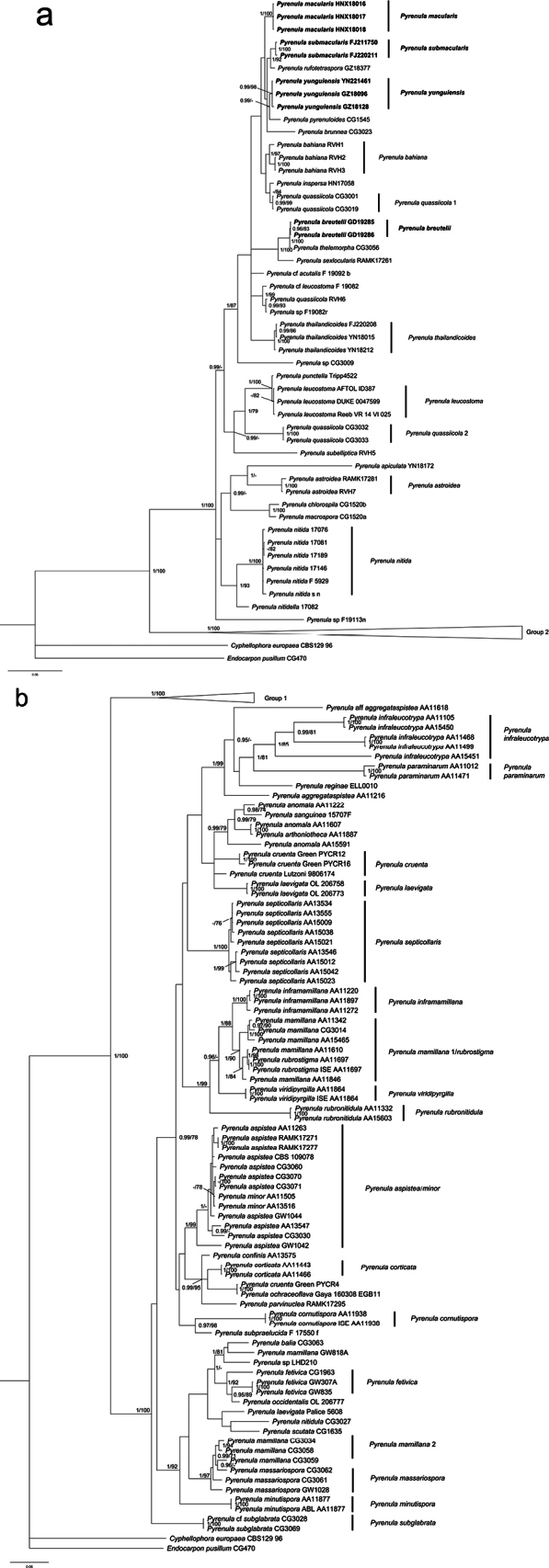
Phylogeny of the genus *Pyrenula* based on a three-gene dataset (mtSSU, ITS and nuLSU) **a** overview of the entire tree and details of Group 1 **b** details of Group 2. Most likely tree obtained using MrBayes. Support values are reported above the branches [posterior probability (PP)/bootstrap value (BS)]. Only significant values (higher than 95% PP and higher than 70% BS) are shown. *Cyphellophoraeuropaea* and *Endocarponpusillum* are the out-group taxa.

The dataset includes 118 ITS sequences, 76 mtSSU sequences and 94 nuLSU sequences, of which 11 ITS sequences, 2 mtSSU sequences, and 9 LSU sequences are newly generated in this study. The BI and ML (Suppl. material 2) trees showed similar topologies, so only the BI tree is provided here as Fig. 1. Compared with the dataset of Gueidan et al. (2016), our phylogenetic analysis includes eleven additional species (the three new species, *Pyrenulamacularis*, *P.breutelii*, *P.sanguinea*, *P.nitidella*, *P.cf.acutalis*, *P.punctella*, *P.cf.leucostoma* and *P.occidentalis*). Our phylogenetic result confirms the presence of two main well-supported monophyletic groups coinciding with the presence/absence of pseudocyphellae as shown in Weerakoon et al. (2012) and Gueidan et al. (2016). Our phylogenetic results also confirm the delimitation problems of several taxa, for example, *P.quassiicola*, *P.mamillana* and *P.rubrostigma*, which is consistent with Gueidan et al. (2016).

The phylogenetic tree revealed five monophyletic lineages corresponding to five different species: *Pyrenulasubmacularis* M.Z. Dou & Z.F. Jia, sp. nov., *P.yunguiensis* M.Z. Dou & Z.F. Jia, sp. nov., *P.rufotetraspora* M.Z. Dou & Z.F. Jia, sp. nov., *P.macularis* (Zahlbr.) R.C. Harris and *P.breutelii* (Müll. Arg.) Aptroot. The clades of the five species were all strongly supported. The support values [posterior probability (PP)/bootstrap value (BS)] of the two specimens of *P.submacularis* was 1/100, the three specimens of *P.yunguiensis* 0.99/98, the two specimens of *P.breutelii* 0.96/83, the three specimens of *P.macularis* 1/100. Although *P.rufotetraspora* clustered with *P.submacularis* with high support (1/92), *P.yunguiensis* clustered with *P.pyrenuloides* (0.99/-), and *P.breutelii* clustered with *P.thelomorpha* (1/100), they can be distinguished easily in anatomical characters. *P.macularis* and *P.breutelii* are far apart on the phylogenetic tree. These five species all belong to Group 1.

### ﻿Chemistry

Before the report (Dou et al. 2024), TLC results had not been described in detail in the literature of *Pyrenula*. They were either not mentioned or interpreted as nothing detected. However, our TLC results show several spots, indicating that there are multiple lichen substances in species of *Pyrenula* (Suppl. materials 3, 4). The weak visibility in short-wave UV (plate A) and the reddish color after charring (plate B) suggest that most spots concern terpenoids. Additional solvents and an experienced chemist will be needed to identify them further. On plate C the terpenoid spots are more intensely colored. A comparison between the *Pyrenula* species represented on the plate C suggests that all have the same series of red color that are green under daylight on the plate B. Reddish spots with yellow rim in class 7 in Suppl. material 3 are probably terpenoids from treebark; they are strongest in samples without lichen. In some samples some of the spots are different from those of the other species.

The TLC experiments of less closely related species were also carried out (Suppl. materials 5, 6). The species in Suppl. materials 5, 6 belong to ten genera (*Pyrenula*, *Phyllobaeis*, *Coenogonium*, *Ocellularia*, *Allographa*, *Graphis*, *Platythecium*, *Phyrrospora*, *Malmidea*, *Thelotrema*) and eight families (Pyrenulaceae, Baeomycetaceae, Coenogoniaceae, Diploschistaceae, Graphidaceae, Lecanoraceae, Malmideaceae, Thelotremataceae). The *Pyrenula* sp. in Suppl. material 5 is a record in China not yet published and included in the Group 2 of phylogenetic tree. This *Pyrenula* species did not produce the chemical substances that were red after charring under 365 nm ultraviolet light and green under daylight. The two white spots of this *Pyrenula* species at Rf 2 on the plate C in Suppl. material 5 might represent the same chemical substances as the two white spots of *P.macularis* (no. nr. 5 and 6) on the plate C in Suppl. material 4. Although the species of *Allographa* (no. nr. 2, 7, 11, 12, 13 of Suppl. material 5) showed red spots after charring under 365 nm ultraviolet light at Rf 5, the spots were yellow under daylight, not green. None of the other species in Suppl. materials 5, 6 produces the same chemical substance as the five species of *Pyrenula* reported here. Given that phylogenetic approach and that chemical characters are underestimated and limited to few specimens and areas, we predict that our findings only represent the tip of the iceberg in this genus.

### ﻿Taxonomy

#### ﻿ Pyrenula submacularis

Taxon classificationFungiVerrucarialesVerrucariaceae

M.Z. Dou & Z.F. Jia sp. nov.

BBBA6B5A-287F-5F79-ABF0-952199DE1984

MycoBank No: 853430

Fig. 2

##### Etymology.

The specific epithet submacularis refers to the similarity to *Pyrenula macularis*.

##### Holotype.

China • Fujian Province, Wuyi Mountain, Tongmu Village Reserve, Wuyi Mountain National Park, Science and Technology Building, 27°44'31"N, 117°40'44"E, alt. 700 m, on bark, 24 October 2021, Y.F. Zhao (LCUF FJ211750, holotype; GenBank PP692372 for ITS, and PP692480 for nuLSU).

##### Diagnosis.

The new species can be distinguished from the most similar species *Pyrenulamacularis* Aptroot by bigger ascospores with more locules and different lichen substances.

##### Description.

Thallus corticolous, crustose, olive-green in the field and khaki after drying, surface dull, corticate with pseudocyphellae, UV-. Apothecia emergent, dispersed, low conical, 1.0–2.0 mm diam., the sides often partly covered by the thallus, with crystals, wall completely carbonized when mature and even falls apart. Ostioles apical, white. Hamathecium not inspersed, IKI+ blue and occasionally red. Ascospores 8 per ascus, uniseriate or subbiseriate, elliptical, with rounded ends, 40–65 × 16–21(–28) μm, hyaline to brown, muriform, with c. 7–9 × 2–7 locules, lumina rounded, old spores containing globules of red oily substance.

##### Chemistry.

Thallus UV-. TLC with solvent C showed unidentified black spots at Rf two, three, four, five and six under 254 nm ultraviolet light; unidentified green spot at Rf four and brick red spots at Rf five after charring under daylight; unidentified red spots at Rf three and four, and one unidentified fluorescent spot at Rf five after charring under 365 nm ultraviolet light (Suppl. material 3).

**Figure 2.**
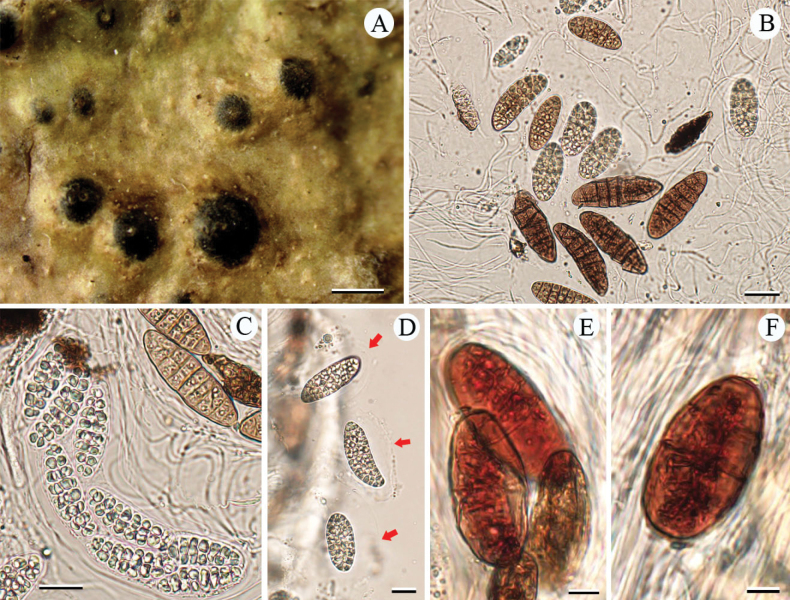
*Pyrenulasubmacularis* sp. nov. (holotype, LCUF FJ211750) **A** thallus with ascomata and pseudocyphellae **B** ascospores at different developmental stages **C** ascus, with 8 ascospores **D** red arrows show gelatinous halo **E, F** over-mature ascospores with orange-oil. Scale bars: 1 mm (**A**); 30 µm (**B**); 20 µm (**C, D**); 10 µm (**E, F**).

##### Habitat and distribution.

The new species is currently only known from the subtropical regions of southern China on bark.

##### Additional specimens examined.

China • Fujian Province, Longyan City, Dongxiao National Forest Park, Frog Stone, 24°58'07"N, 117°01'14"E, alt. 679 m, on bark, 12 July 2022, Z.G. Ma (LCUF FJ220211; GenBank PP692377 for ITS, and PP692481 for nuLSU).

##### Notes.

This new species is similar to *Pyrenulaseminuda*, *P.endocrocea*, *P.breutelii* and *P.macularis* in having (sub)muriform ascospores with red or orange oil when over-mature. This new species differs from *P.seminuda* by bigger and muriform ascospores with more locules, the latter 22–40 × 10–17 μm and submuriform with 6 × 1–2 locules, most transverse locules being single and few with an oblique or longitudinal division (Aptroot et al. 2013). *P.endocrocea* differs from this new species by medulla with a soft layer of copious orange anthraquinone crystals reacting UV+ red and KOH+ crimson, and smaller ascospores, (30–)32–44(–50) × 13–16(–19) μm (Aptroot et al. 2012). This new species (*P.submacularis*) differs from *P.breutelii* in lichen substances (Suppl. material 3) and by bigger ascospores and more locules, the latter 25–35 × 12–13 μm and 8 × 3–4 locules (Müller 1885). *P.submacularis* (no. nr. 5 and nr. 6 in Suppl. material 3) has two more black spots at Rf two and three under 254 nm ultraviolet light than *P.breutelii* (no. nr. 15 and nr. 16 in Suppl. material 3). And the new species has one more spot with fluorescence at Rf five after charring under 365 nm ultraviolet light than *P.breutelii* (Suppl. materials 3, 4). This new species (*P.submacularis*) can be distinguished from the most similar species *P.macularis* by different lichen substances and bigger ascospores and more locules, the latter 34–45 × 14–16 μm and 8 × 1–3 locules (Zahlbruckner 1930). *P.macularis* (no. nr. 5 and nr. 6 in Suppl. material 4) has two more black spots at Rf two under 254 nm ultraviolet light than *P.submacularis* (no. nr. 8 and nr. 10 in Suppl. material 4). And the new species has one more spot with fluorescence at Rf five after charring under 365 nm ultraviolet light than *P.macularis* (Suppl. materials 3, 4).

#### ﻿ Pyrenula yunguiensis

Taxon classificationFungiVerrucarialesVerrucariaceae

M.Z. Dou & Z.F. Jia sp. nov.

34317F30-C601-5AF3-B029-CAA50E65C988

MycoBank No: 853431

Fig. 3

##### Etymology.

The specific epithet yunguiensis refers to the place where the specimen was collected.

##### Holotype.

China • Guizhou Province, Duyun City, Doupeng Mountain Reserve, Mayao River Street, 26°22'32"N, 107°22'11"E, alt. 1107 m, on bark, 17 March 2018, F.Y. Liu (LCUF GZ18096, holotype; GenBank PP692374 for ITS, and PP692478 for nuLSU).

##### Diagnosis.

The new species can be distinguished from the most similar species *P.submacularis* by bigger ascospores and different lichen substances.

##### Description.

Thallus corticolous, crustose, olive-green in the field and khaki after drying, surface dull, corticate with pseudocyphellae, UV-. Apothecia emergent, dispersed, low conical, 0.5–2.0 mm diam., the sides often partly covered by the thallus, with crystals. Excipulum carbonized when mature and falls apart when over-mature. Ostioles apical, white. Hamathecium not inspersed, IKI+ red and occasional blue, the colour relating to development stage. Ascospores 8 per ascus, uniseriate, fusiform, with pointed or blunt ends, 50–70(–80) × 17–22(–26) μm, hyaline to brown, muriform, with c. 8 × 2–4 locules, lumina rounded, old spores containing globules of red oily substance.

**Figure 3.**
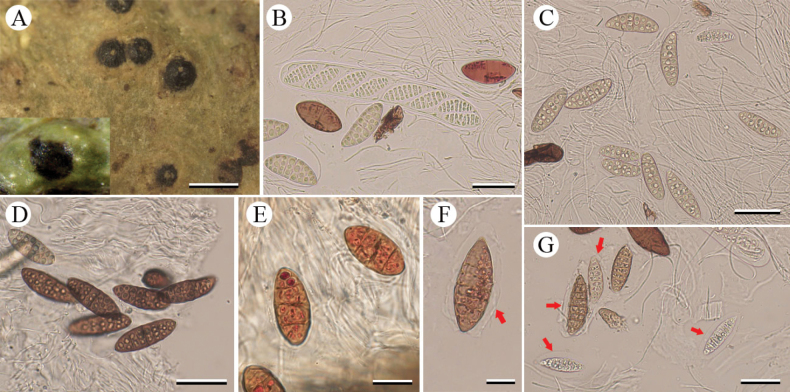
*Pyrenulayunguiensis* sp. nov. (holotype, LCUF GZ18096) **A** thallus with ascomata and pseudocyphellae **B** ascus, with 8 ascospores **C–G** ascospores at different developmental stages **E, F** over-mature ascospores with red-oil **F, G** red arrows show gelatinous halo. Scale bars: 1 mm (**A**); 50 µm (**B–D**); 20 µm (**E, F**); 40 µm (**G**).

##### Chemistry.

Thallus UV-. TLC with solvent C showed unidentified black spots at Rf four and six under 254 nm ultraviolet light; unidentified green spot at Rf four on charred plate under daylight; unidentified red spots at Rf three, four and five on charred plate under 365 nm ultraviolet light (Suppl. material 3).

##### Habitat and distribution.

The new species is currently only known from the subtropical regions of southern China on bark.

##### Additional specimens examined.

China • Guizhou Province, Duyun City, Doupeng Mountain Reserve, Old Post Street, 26°22'35"N, 107°21'52"E, alt. 1154 m, on bark, 17 March 2018, X.H. Wu (LCUF GZ18128; GenBank PP692373 for ITS and PP692479 for nuLSU). China • Yunnan Province, Jingdong County, Taizhong Town, Aishanting, 24°32'11"N, 101°01'53"E, alt. 2625 m, on bark, 16 August 2022, T. Jia (LCUF YN221461; GenBank PP692378 for ITS, and PP692477 for nuLSU).

##### Notes.

This new species is similar to *Pyrenulaseminuda*, *P.endocrocea*, *P.breutelii*, *P.macularis* and *P.submacularis* in having (sub)muriform ascospores with red or orange oil when over-mature. This new species differs from *P.seminuda* by bigger and muriform ascospores with more locules, the latter 22–40 × 10–17 μm and submuriform with 6 × 1–2 locules, most transverse locules being single and few with an oblique or longitudinal division (Aptroot et al. 2013). *P.endocrocea* differs from this new species by medulla with a soft layer of copious orange anthraquinone crystals reacting UV+ red and KOH+ crimson, and smaller ascospores, (30–)32–44(–50) × 13–16(–19) μm (Aptroot et al. 2012). This new species differs from *P.breutelii* by bigger ascospores, the latter 25–35 × 12–13 μm (Müller 1885). This new species can be distinguished from *P.macularis* by bigger ascospores, the latter 34–45 × 14–16 μm and 8 × 1–3 locules (Zahlbruckner 1930). This new species can be distinguished from the most similar species *P.submacularis* by different lichen substances, bigger ascospores and less locules, the latter 40–65 × 16–21(–28) μm and 7–9 × 2–7 locules. *P.submacularis* (no. nr. 5 and nr. 6 in Suppl. material 3; no. nr. 8 and nr. 10 in Suppl. material 4) has one more spot with fluorescence at Rf five after charring under 365 nm ultraviolet light than *P.yunguiensis* (no. nr. 8 and nr. 11 in Suppl. material 3; no. nr. 12 and nr. 14 in Suppl. material 4). Although *P.yunguiensis* clustered with *P.pyrenuloides* with high support (0.99/-), they can be distinguished easily in anatomical characters. *P.pyrenuloides* has no red or orange oil in over-mature ascospores and more locules (8–10 rows of up to ca. 10 each) (Harris 1989).

#### ﻿ Pyrenula rufotetraspora

Taxon classificationFungiVerrucarialesVerrucariaceae

M.Z. Dou & Z.F. Jia sp. nov.

19BDA554-27BB-59E3-8489-A687A7CAABB3

MycoBank No: 853432

Fig. 4

##### Etymology.

The specific epithet rufo refers to the red oil in over-mature ascospores and tetraspora means that there are four spores in each ascus.

##### Holotype.

China • Guizhou Province, Libo County, Xiaoqikong Scenic Area, Laya Waterfall, 25°15'10"N, 107°44'06"E, alt. 425 m, on bark, 24 October 2018, Z.F. Jia (LCUF GZ18377, holotype; GenBank PP692371 for ITS, and PP692474 for nuLSU).

##### Diagnosis.

This new species can be distinguished from the most similar species *Pyrenulayunguiensis* by fewer ascospores per ascus, bigger ascospores, more locules and different lichen substances.

##### Description.

Thallus corticolous, crustose, olive-green in the field and khaki after drying, surface dull, corticate with pseudocyphellae, UV-. Apothecia emergent, dispersed, conical, 0.6–1.2 mm diam., the sides often partly covered by the thallus, with crystals. Excipulum completely carbonized when mature and falls apart when over-mature. Ostioles apical, white or brown. Hamathecium not inspersed, IKI+ red. Ascospores 4 per ascus, uniseriate, fusiform, with pointed or blunt ends, 70–100(–106) × (17–)21–27(–41) μm, hyaline to brown, muriform, with c. 10–12 × 3–14 locules, lumina rounded, old spores containing globules of red oily substance.

**Figure 4.**
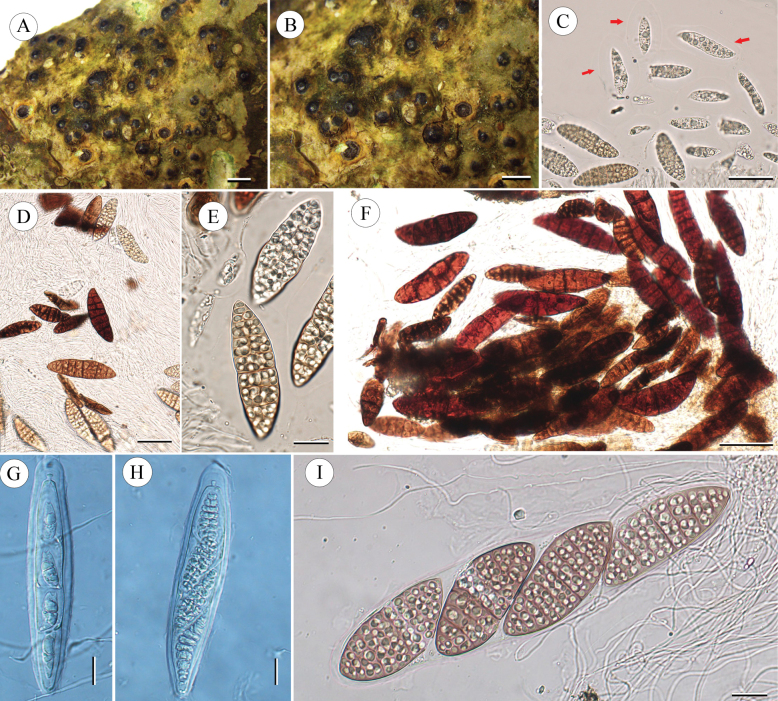
*Pyrenularufotetraspora* sp. nov. (holotype, LCUF GZ18377) **A, B** thallus with ascomata **C–F** ascospores at different developmental stages, red arrows in **C** show gelatinous halo around immature ascospores **G–I** ascus, with 4 ascospores. Scale bars: 2 mm (**A, B**); 50 µm (**C, D**); 20 µm (**E**); 50 µm (**F**); 20 µm (**G, H**); 25 µm (**I**).

##### Chemistry.

Thallus UV-. TLC with solvent C showed one unidentified black spot at the dividing line of Rf three and four under 254 nm ultraviolet light; unidentified red substances at Rf four under 365 nm ultraviolet light (Suppl. material 3).

##### Habitat and distribution.

The new species is currently only known from the subtropical regions of southern China on bark.

##### Notes.

This new species is similar to *Pyrenulaseminuda*, *P.endocrocea*, *P.breutelii*, *P.macularis*, *P.submacularis* and *P.yunguiensis* in having (sub)muriform ascospores with red or orange oil when over-mature. This new species differs from *P.seminuda* by bigger and muriform ascospores with more locules, the latter 22–40 × 10–17 μm and submuriform with 6 × 1–2 locules, most transverse locules being single and few with an oblique or longitudinal division (Aptroot et al. 2013). *P.endocrocea* differs from this new species by medulla with a soft layer of copious orange anthraquinone crystals reacting UV+ red and KOH+ crimson, and smaller ascospores, (30–)32–44(–50) × 13–16(–19) μm (Aptroot et al. 2012). This new species can be distinguished from *P.breutelii*, *P.macularis*, *P.submacularis* and *P.yunguiensis* by different lichen substances (Suppl. materials 3, 4), bigger ascospores, more locules and fewer ascospores per ascus. There are 8 ascospores in per ascus in *P.breutelii*, *P.macularis*, *P.submacularis* and *P.yunguiensis*, 4 in the new species. *P.rufotetraspora* showed a black spot at the dividing line of Rf three and four under 254 nm ultraviolet light (no. nr. 13 on Suppl. material 3), which was not red on charred plate under 365 nm ultraviolet light. This black spot did not exist in *P.breutelii*, *P.macularis*, *P.submacularis* and *P.yunguiensis* and located at Rf four under 254 nm ultraviolet light on Suppl. material 4 (no. nr. 16). The difference of locations of this spot on Suppl. materials 3, 4 might be caused by edge effect. *P.submacularis* is sister to *P.rufotetraspora* with high support (1/92), but the latter has fewer ascospores in ascus (4) and obviously bigger ascospores.

#### ﻿ Pyrenula breutelii

Taxon classificationFungiVerrucarialesVerrucariaceae

(Müll. Arg.) Aptroot

5BD62299-99C3-5145-90BA-2B5EA905AA1E

MycoBank No: 563102

Fig. 5

##### Basionym.

*Anthracotheciumbreutelii* Müll. Arg., *Flora* 68: 339 (1885).

##### Holotype.

St Thomas, *Breutel*, ex hb. Hampe 1877 (G).

##### Description.

Thallus corticolous, crustose, olive-green in the field and khaki after drying, surface dull, corticate with abundant pseudocyphellae, UV-. Apothecia perithecioid, dispersed, aggregated occasionally when crowded, low conical, 0.3–0.5 mm diam., the sides often partly covered by the thallus, with crystals. Excipulum carbonized when mature and falls apart when over-mature. Ostioles apical, white. Hamathecium not inspersed, IKI+ red. Ascospores 8 per ascus, subbiseriate, fusiform, with pointed or blunt ends, (23–)25–37(–41) × (10–)12–15(–18) μm, hyaline to brown, muriform, with c. 8 × 1–4 locules, lumina rounded, old spores containing globules of red oily substance.

**Figure 5.**
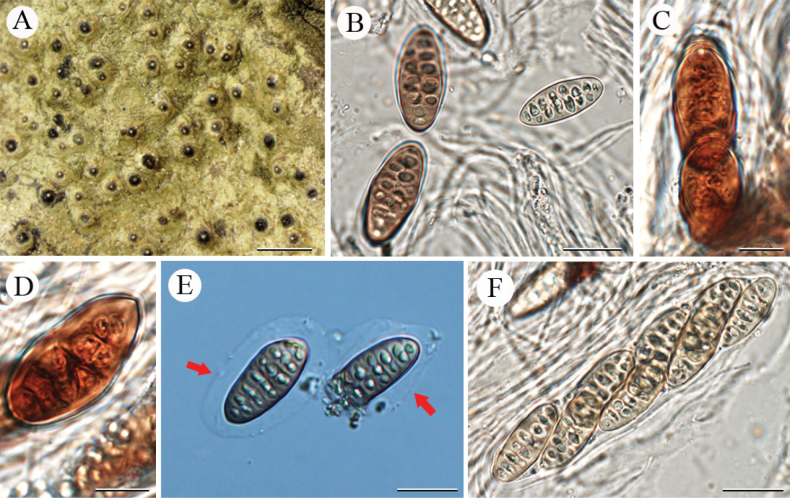
*Pyrenulabreutelii* (LCUF GD19285) **A** thallus with ascomata and pseudocyphellae **B** hyaline immature ascospores and brown mature ascospores **C, D** over-mature ascospores with red oil **E** red arrows show gelatinous halo **F** ascus, with 8 ascospores. Scale bars: 1 mm (**A**); 20 µm (**B**); 10 µm (**C, D**); 20 µm (**E, F**).

##### Chemistry.

Thallus UV-. TLC with solvent C showed unidentified black spots at Rf four and five under 254 nm ultraviolet light on fresh plate; unidentified green spot at Rf four on charred plate under daylight; unidentified red spots at Rf three, four and five, unidentified red and black spots at Rf five on charred plate under 365 nm ultraviolet light (Suppl. material 3).

##### Additional specimens examined.

China • Guangdong Province, Guangzhou City, South China Botanical Garden, Chinese Academy of Sciences, Australian Garden, Energy Road, 23°10'44"N, 113°21'20"E, alt. 26 m, on bark, 20 January 2019, Z.T. Yao (LCUF GD19285; GenBank PP692375 for ITS and PP692475 for LSU). China • Guangdong Province, Guangzhou City, South China Botanical Garden, Chinese Academy of Sciences, Australian Garden, Energy Road, 23°10'44"N, 113°21'20"E, alt. 26 m, on bark, 20 January 2019, Z.T. Yao (LCUF GD19286; GenBank PP692376 for ITS, PP692476 for nuLSU and PP659692 for mtSSU).

##### Habitat and distribution.

Growing on tree bark of pantropical forests. Previously reported from the U.S.A (Müller 1885). Newly reported for China.

##### Notes.

The morphology and anatomy characteristics of the Chinese specimens correspond to *Pyrenulabreutelii* (Müll. Arg.) Aptroot described from St Thomas, *Breutel*, ex hb. Hampe. *Pyrenula*macularis is distinguished by larger ascomata (0.3–1.5 mm), larger ascospores (35–45 × 14–16) μm and less locules (1–3) (Zahlbruckner 1930). In the protolog, TLC and KI result was not mentioned and molecular sequences were not provided. Here, we provide TLC, KI result and ITS and nuLSU sequences. Because the difference in ascospores between *P.macularis* and *P.breutelii* is not very significant, *P.macularis* was synonymized with *P.breutelii* (Aptroot 2012; Aptroot et al. 2013). But the phylogenetic result and TLC result proves they are two different species. This is the first report of *P.breutelii* in China. Although *P.breutelii* clustered with *P.thelomorpha* with high support (1/100), they can be distinguished easily in anatomical characters. *P.thelomorpha* has no red or orange oil in over-mature ascospores and more locules (8 rows of c. 3–8 locules) (Aptroot 2009).

#### ﻿ Pyrenula macularis

Taxon classificationFungiVerrucarialesVerrucariaceae

(Zahlbr.) R.C. Harris

42AC117B-B853-54F4-A6BD-FC9B0E0FCDB9

MycoBank No: 134429

Fig. 6

##### Basionym.

*Anthracotheciummaculare* Zahlbr., *Mycologia* 22: 70 (1930).

##### Holotype.

Yauco, Porto Rico [Puerto Rico], 30 Dec. 1915.

##### Description.

Thallus corticolous, crustose, olive-green in the field and khaki after drying, surface dull, corticate with abundant pseudocyphellae, UV-. Apothecia perithecioid, conical, dispersed, aggregated occasionally when crowded, with crystals, immersed in the thallus, small, to 0.3 mm wide at the early developmental stage, then the sides partly covered by the thallus. Excipulum carbonized when mature and falls apart when over-mature. Ostioles apical, white. Hamathecium not inspersed, IKI+ red. Ascospores 8 per ascus, subbiserial, fusiform, with pointed or blunt ends, (33–)37–50 × (13–)14–16 μm, hyaline to brown, muriform, with c. 8 × 1–3 locules, lumina rounded, old spores containing globules of red oily substance.

**Figure 6.**
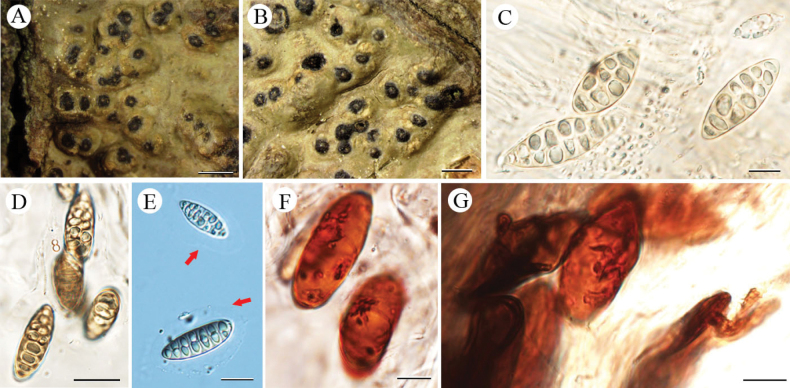
*Pyrenulamacularis* (LCUF HNX18017) **A, B** thallus with ascomata and pseudocyphellae **C, D** ascospores at different development stages **E** ascospores with gelatinous halo shown by red arrows **F, G** over-mature ascospores with orange oil. Scale bars: 1 mm (**A, B**); 10 µm (**C**); 20 µm (**D, E**); 10 µm (**F, G**).

##### Chemistry.

Thallus UV-. TLC with solvent C showed unidentified black spots at Rf two, four and five under 254 nm ultraviolet light on fresh plate; unidentified green spot at the dividing line of Rf four and five on charred plate under daylight; unidentified red spots at Rf three, four and red spots at Rf five on charred plate under 365 nm ultraviolet light (Suppl. material 4).

##### Additional specimens examined.

China • Hunan Province, Wugang City, Yun Shan, Shuanghua Pavilion, 26°39'28"N, 110°36'37"E, alt. 730 m, on bark of *Zelkovaserrata*, 28 April 2018, Z.F. Jia (LCUF HNX18016; GenBank PP692368 for ITS). China • Hunan Province, Wugang City, Yun Shan, Shuanghua Pavilion, 26°39'28"N, 110°36'37"E, alt. 730 m, on bark of *Zelkovaserrata*, 28 April 2018, Z.F. Jia (LCUF HNX18017; GenBank PP692369 for ITS). China • Hunan Province, Wugang City, Yun Shan, Shuanghua Pavilion, 26°39'28"N, 110°36'37"E, alt. 730 m, on bark of *Zelkovaserrata*, 28 April 2018, Z.F. Jia (LCUF HNX18018; GenBank PP659691 for mtSSU, PP692370 for ITS and PP692473 for nuLSU).

##### Habitat and distribution.

Growing on exposed tree in pantropical forest. Previously reported from Porto Rico (Zahlbruckner 1930), U.S.A. (Harris 1989), Australia (Aptroot 2009), Puntarenas (Aptroot et al. 2008), Muri Lagoon (Mccarthy 2000), HongKong (Aptroot and Seaward 1999) and Taiwan (Aptroot 2003) of China.

##### Notes.

The morphology and anatomy characteristics of the Chinese specimens correspond to *Pyrenulamacularis* (Zahlbr.) R.C. Harris described from Yauco, Porto Rico. *P.breutelii* is distinguished by different lichen substances (Suppl. material 4), smaller ascomata (0.3–0.5 mm), smaller ascospores ((23–)25–37(–41) × (10–)12–15(–18)) μm and more locules (1–4). *P.macularis* (no. nr. 5 and 6) has two more black spots at Rf two under 254 nm ultraviolet light on fresh plate than *P.breutelii* (no. nr. 2 and 3) (Suppl. material 4). In the protolog, TLC and KI result was not mentioned and molecular sequences were not provided. Here, we provide TLC, KI result and ITS, nuLSU sequences. This species has previously been reported in China only in Hong Kong (Aptroot and Seaward 1999) and Taiwan (Aptroot 2003). Because the difference in ascospores between *P.macularis* and *P.breutelii* is not very significant, *P.macularis* was synonymous with *P.breutelii* (Aptroot 2012; Aptroot et al. 2013). But the phylogenetic result and TLC results prove they are two different species.

## ﻿Discussion

Because the differences in ascospores between *Pyrenulamacularis* and *P.breutelii* are not very significant, *P.macularis* was considered synonymous with *P.breutelii* (Aptroot 2012; Aptroot et al. 2013). But both the phylogenetic and the TLC results prove they are two different species. Similarly, if without molecular phylogenetic analysis and rigorous TLC trials, *P.submacularis* M.Z. Dou & Z.F. Jia, sp. nov. and *P.yunguiensis* M.Z. Dou & Z.F. Jia, sp. nov. might be classified into the same species, and *P.submacularis* M.Z. Dou & Z.F. Jia, sp. nov. might be classified as *P.macularis*. It is obvious that phylogenetic analysis and metabolite detection are crucial in the taxonomic study of *Pyrenula*, but they have been thus far limited to very few specimens, which may partly explain the delimitation problems in *P.quassiicola*, *P.mamillana*, *P.minor*, *P.aspistea* and other taxa revealed in previous and our phylogenetic analysis of this genus (Gueidan et al. 2016; Weerakoon et al. 2012). The delimination of the five species in this study suggests that it is significant to pay attention to the chemical substances in distinguishing phylogenetically informative characters and revealing near-cryptic diversification (Lücking et al. 2021) of *Pyrenula*.

Meanwhile, I carried out the TLC experiments of some remote species as Suppl. materials 5, 6. The *Pyrenula* sp., which was one new record in China unpublished and included in the Group 2 of phylogenetic tree, did not produce the same set of terpenoids as the five species described in this article. But it showed two white spots at Rf 2 on the plate C, which might represent the same chemical substances as that *P.macularis* produced. The other species were included in other families, and did not produce the same chemicals as the five species. This seems to suggest that the likelihood of producing the same chemical is positively related to the distance of phylogenetic relationships. In Southern China, there are abundant subtropical to tropical evergreen resources (Zhu 2017). This habitat is favorable for the pyrenocarpous lichens, including *Pyrenula*. However, the genus has not been sufficiently studied in China. Most of *Pyrenula* reported in China were in the checklist of the lichens of Hong Kong and Taiwan (Aptroot and Seaward 1999; Aptroot 2003). Furthermore, the vast majority of *Pyrenula* reported in China were new records and had no molecular data. Our research suggests high species richness of *Pyrenula* is expected to be found when the taxonomic studies of this genus were carried out systematically in China.

### ﻿Key to the species of *Pyrenula* reported in China

Key A (Ascospores submuriform to muriform).

Key B (Ascospores only transversely septate).

#### ﻿Key A

**Table:** 

1	Thallus yellow to orange; anthraquinones pigments K+ pink to purplish	***Pyrenulaochraceoflava* (Nyl.) R.C. Harris**
–	Thallus K– or yellowish, anthraquinones absent	**2**
2	Ostioles lateral	**3**
–	Ostioles apical	**4**
3	Ascospores < 70 μm long	***Pyrenulaastroidea* (Fée) R.C. Harris**
–	Ascospores > 70 μm long	***Pyrenulaschiffneri* (Zahlbr.) Aptroot**
4	Ascospores < 25 μm long	**5**
–	Ascospores > 25 μm long	**6**
5	Thallus UV+yellow	***Pyrenulaconfinis* (Nyl.) R.C. Harris**
–	Thallus UV	***Pyrenulaparvinuclea* (Meyen & Flot.) Aptroot**
6	Old ascospores with orange oil	**7**
–	Old ascospores without orange oil	**11**
7	Ascospores 4/ascus, 70–100(–106) × (17–)21–27(–41) μm, 10–12 × 3–14 locules	***Pyrenularufotetraspora* M.Z. Dou & Z.F. Jia, sp. nov.**
–	Ascospores 8/ascus	**8**
8	TLC with solvent C showed one spot with fluorescence at Rf five under 365 nm ultraviolet light, ascospores 40–65 × 16–21(–28) um, 7–9 × 2–7 locules	***Pyrenulasubmacularis* M.Z. Dou & Z.F. Jia, sp. nov.**
–	TLC with solvent C showed no fluorescent spot at Rf five under 365 nm ultraviolet light	**9**
9	Most ascospores < 50 μm long	**10**
–	Ascospores 50–70(–80) × 17–22(–26) μm, 8 × 2–4 locules	***Pyrenulayunguiensis* M.Z. Dou & Z.F. Jia, sp. nov.**
10	Ascospores (23–)25–37(–41) × (10–)12–15(–18) μm, 8 × 1–4 locules, TLC with solvent C showed no black spot at Rf two under 254 nm ultraviolet light	***Pyrenulabreutelii* (Müll. Arg.) Aptroot**
–	Ascospores (33–)37–50 × (13–)14–16 μm, 8 × 1–3 locules, TLC with solvent C showed two black spots at Rf two under 254 nm ultraviolet light	***Pyrenulamacularis* (Zahlbr.) R.C. Harris**
11	Ascospores > 80 μm long, mostly 2/ascus	**12**
–	Ascospores < 80 μm long, mostly 4–8/ascus	**13**
12	Thallus without pseudocyphellae, ascospores 80–140(–155) μm long	***Pyrenulaplatystoma* (Müll. Arg.) Aptroot**
–	Thallus with pseudocyphellae, ascospores 115–180 μm long	***Pyrenuladuplicans* (Nyl.) Aptroot**
13	Locules relatively large and angular, with up to 6 between 2 primary septa	***Pyrenulaleucostoma* Ach.**
–	Locules mostly round, at least in the central part of the ascospore with more than 6 between 2 primary septa	***Pyrenulapyrenuloides* (Mont.) R.C. Harris**

#### ﻿Key B

**Table:** 

1	Ostioles pointing in various directions, mostly eccentric to lateral; ascomata sometimes with several chambers connected to joint ostioles	**2**
–	Ostioles apical or, when eccentric, all pointing in the same direction; ascomata with one chamber or each chamber with own ostiole	**4**
2	Terminal locules directly against the exospore wall; ascospores 16–25 μm long	***Pyrenulacircumfiniens* Vain.**
–	Terminal locules separated from the exospore wall by endospore thickening	**3**
3	Ascospores 35–45 μm long	***Pyrenulaadacta* Fée**
–	Ascospores 18–30 µm long	***Pyrenulaacutispora* Kalb & Hafell**
4	Ascospores at least seemingly 4–7-septate; old ascospores with orange oil, thallus often with pseudocyphellae	***Pyrenulasexlocularis* (Nyl.) Müll. Arg.**
–	Ascospores all distinctly 3-septate	**5**
5	Ascospores (45–)50–60 µm long, thallus without papillae but with pseudocyphellae	***Pyrenulaimmissa* (Stirt.) Zahlbr.**
–	Ascospores mostly < 50 µm long	**6**
6	Ascomata erumpent, c. 0.4–0.8 mm diam; thallus with red patches or completely red; ascospores 27–35 µm long	***Pyrenulacruenta* (Mont.) Vain.**
–	Ascomata and thallus without external pigments	**7**
7	Ascomata mostly aggregated, with fused walls but with separate ostioles	**8**
–	Ascomata mostly simple, only aggregated as by chance when crowded	**10**
8	Old ascospores with red oil; hamathecium inspersed, ascospores 28.5–50 × 10–20 μm	***Pyrenulainspersa* M.Z. Dou & Z.F. Jia**
–	Old ascospores without red oil	**9**
9	Ascospores mostly 21–25 µm long	***Pyrenulaleucotrypa* (Nyl.) Upreti**
–	Ascospores mostly 15–20 µm long	***Pyrenulaanomala* (Ach.) Vain.**
10	Old ascospores with orange oil	**11**
–	Old ascospores without orange oil	**14**
11	Ascospores < 35 μm long	**12**
–	Ascospores > 35 μm long	**13**
12	Terminal locules directly against the exospore wall	***Pyrenulaapiculata* M.Z. Dou & Z.F. Jia**
–	Terminal locules separated from the exospore wall by endospore thickening	***Pyrenulabahiana* Malme**
13	Hamathecium IKI-; no substances detected by TLC	***Pyrenulathailandica* Aptroot**
–	Hamathecium IKI+ red; TLC showed an unidentified spot at Rf four under 254 nm ultraviolet light using solvent C	***Pyrenulathailandicoides* M.Z. Dou & Z.F. Jia**
14	Terminal locules all directly against the exospore wall	**15**
–	Terminal locules mostly (at least in mature ascospores) separated from the exospore wall by endospore thickening	**19**
15	Thallus UV+ yellow	***Pyrenulapseudobufonia* (Rehm) R.C. Harris**
–	Thallus UV	**16**
16	Hamathecium inspersed	**17**
–	Hamathecium not inspersed	***Pyrenulanitidula* (Bres.) R.C. Harris**
17	Ascospores all < 16 µm long	***Pyrenulacayennensis* Müll. Arg.**
–	Ascospores partly > 16 µm long	**18**
18	Hamathecium inspersed only in the upper part	***Pyrenulaacutalis* R.C. Harris**
–	Hamathecium totally inspersed	***Pyrenulafetivica* (Krempelh.) Müll. Arg**
19	Hamathecium inspersed	**20**
–	Hamathecium not inspersed	**24**
20	Central ascospore locules elongated	***Pyrenulasubelliptica* (Tuck.) R.C. Harris**
–	Central ascospore locules transversely lenticular to rounded	**21**
–	Ascomata mostly < 0·7 mm diam; ascospores 18–20 µm long; thallus and ascomata without any anthraquinone	***Pyrenulasubglabrata* (Nyl.) Müll. Arg.**
–	Ascomata mostly > 0·7 mm diam	**22**
22	Ascospores mostly 10–20 µm long	***Pyrenulamamillana* (Ach.) Trevis.**
–	Ascospores mostly > 20 µm long	**23**
23	Ascospores rounded, uniseriate in the ascus	***Pyrenulamassariospora* (Starb.) R.C. Harris**
–	Ascospores at least at one end pointed, biseriate in the ascus	***Pyrenulaacutalis* R.C. Harris**
24	Thallus UV+yellow	***Pyrenuladermatodes* (Borrer) Schaer**
–	Thallus UV− or greenish/whitish reflecting	**25**
25	Ascospores mostly > 25 µm long	**26**
–	Ascospores mostly < 25 µm long	**30**
26	Ascospores 36–45 µm long, without black granules at the tips	***Pyrenulasubducta* (Nyl.) Müll. Arg.**
–	Ascospores < 40 µm long, without black granules at the tips	**27**
27	Ascomata mostly > 0·7 mm diam	***Pyrenulacomplanata* (Mont.) Trevis.**
–	Ascomata mostly < 0·7 mm diam	**28**
28	Ascospores 32–42 µm long	***Pyrenulapunctella* (Nyl.) Trevis.**
–	Ascospores mostly 25–37 µm long	**29**
29	Central locules much wider than long, ascomata conical, emergent, thallus without pseudocyphellae	***Pyrenulamastophora* (Nyl.) Müll. Arg.**
–	Central locules more or less rounded, ascomata somewhat rounded, often partly immersed in the thallus, thallus often with pseudocyphellae	***Pyrenulaquassiaecola* (Fée) Fée**
30	Ascospores mostly 21–25 µm long	**31**
–	Ascospores mostly < 21 µm long	**34**
31	Ascomata with red, KOH+ purple crystals inside; ascomata > 0·5 mm diam	***Pyrenulanitida* (Weigel) Ach.**
–	Ascomata without red crystals	**32**
32	Ascospores with angular diamond-shaped locules	***Pyrenulamicheneri* R.C. Harris**
–	Ascospores with rounded or quadrangular locules	**33**
33	Ascospores with at least one pointed end	***Pyrenulaacutispora* Kalb & Hafellner**
–	Ascospores with rounded ends	***Pyrenulasubmastophora* Ajay Singh & Upreti**
34	Ascospores mostly < 15 µm long	**35**
–	Ascospores mostly > 15 µm long	**36**
35	Ascospores 6–8 µm wide	***Pyrenulabrunnea* Fée**
–	Ascospores 4–6 µm wide	***Pyrenulaaspistea* (Ach.) Ach**
36	Ascomata > 0·7 mm diam	***Pyrenulascutata* (Stirt.) Zahlbr.**
–	Ascomata < 0·7 mm diam	**37**
37	Ascospores with dark bands between the locules	***Pyrenulaconfoederata* R.C. Harris**
–	Ascospores without dark bands	***Pyrenulaaggregata* (Fée) Fée**

## Supplementary Material

XML Treatment for Pyrenula submacularis

XML Treatment for Pyrenula yunguiensis

XML Treatment for Pyrenula rufotetraspora

XML Treatment for Pyrenula breutelii

XML Treatment for Pyrenula macularis

## ﻿Additional information

### Author ORCIDs

Mingzhu Dou https://orcid.org/0000-0001-5160-1517

Jiechen Li https://orcid.org/0009-0001-8819-8407

Yongshun Hu https://orcid.org/0009-0002-6764-4364

André Aptroot https://orcid.org/0000-0001-7949-2594

### Data availability

Publicly available datasets were analyzed in this study. All resulting alignments were deposited in TreeBASE (http://purl.org/phylo/treebase/phylows/study/TB2:S31341). All newly generated sequences were deposited in GenBank (https://www.ncbi.nlm.nih.gov/genbank/ (accessed on 31 December 2025). All new taxa were deposited in MycoBank (https://www.mycobank.org/ (accessed on 30 June 2025)).

## Supplementary materials

Supplementary material 1 Information for the sequences download from NCBI used in this study Data type docx https://binary.pensoft.net/file/1173210This dataset is made available under the Open Database License (http://opendatacommons.org/licenses/odbl/1.0/). The Open Database License (ODbL) is a license agreement intended to allow users to freely share, modify, and use this Dataset while maintaining this same freedom for others, provided that the original source and author(s) are credited.Mingzhu Dou

Supplementary material 2 Phylogenetic tree constructed through ML analyses based on mtSSU, ITS, and nuLSU for *Pyrenula* Data type pdf Explanation note *Cyphellophoraeuropaea* and *Endocarponpusillum* are the out-group taxa. Only significant values (higher than 70% BS) are shown. https://binary.pensoft.net/file/1173211This dataset is made available under the Open Database License (http://opendatacommons.org/licenses/odbl/1.0/). The Open Database License (ODbL) is a license agreement intended to allow users to freely share, modify, and use this Dataset while maintaining this same freedom for others, provided that the original source and author(s) are credited.Mingzhu Dou

Supplementary material 3 TLC test using C solvent system Data type jpg Explanation note **a**: fresh plate under 254 nm ultraviolet light; **b**: charring plate under daylight; **c**: charring plate under 365 nm ultraviolet light. 0: standard substance (*Lethariellacladonioides*, norstictic acid at Rf four and atranorin at Rf seven). 1: Bark without thallus of HN18017; 2: HN18017 (*Pyrenulamacularis*); 3: HN18018 (*P.macularis*). 4: Bark without thallus of FJ211750; 5: FJ211750 (*P.submacularis*); 6: FJ220211 (*P.submacularis*). 7: Bark without thallus of GZ18096; 8: GZ18096 (*P.yunguiensis*); 9: Other taxon; 10. Bark without thallus of YN221461; 11. YN221461 (*P.yunguiensis*). 12: Bark without thallus of GZ18377; 13: GZ18377 (*P.rufotetraspora*). 14: Bark without thallus of GD19285; 15: GD19285 (*P.breutelii*); 16: GD19286 (*P.breutelii*). https://binary.pensoft.net/file/1173212This dataset is made available under the Open Database License (http://opendatacommons.org/licenses/odbl/1.0/). The Open Database License (ODbL) is a license agreement intended to allow users to freely share, modify, and use this Dataset while maintaining this same freedom for others, provided that the original source and author(s) are credited.Mingzhu Dou

Supplementary material 4 TLC test using C solvent system Data type jpg Explanation note **a**: fresh plate under 254 nm ultraviolet light; **b**: charring plate under daylight; **c**: charring plate under 365 nm ultraviolet light. The spots visible in 254 nm UV on the fresh plate are marked with pencil. 0: standard substance (*Lethariellacladonioides*, norstictic acid at Rf four and atranorin at Rf seven). 1: Bark without thallus of GD19285; 2: GD19285 (*Pyrenulabreutelii*); 3: GD19286 (*P.breutelii*). 4: Bark without thallus of HNX18017; 5: HNX18017 (*P.macularis*); 6: HNX18018 (*P.macularis*). 7: Bark without thallus of FJ211750; 8: FJ211750 (*P.submacularis*); 9: Bark without thallus of FJ220211; 10: FJ220211 (*P.submacularis*). 11: Bark without thallus of GZ18096; 12: GZ18096 (*P.yunguiensis*); 13. Bark without thallus of YN221461; 14. YN221461 (*P.yunguiensis*). 15: Bark without thallus of GZ18377; 13: GZ18377 (*P.rufotetraspora*). https://binary.pensoft.net/file/1173213This dataset is made available under the Open Database License (http://opendatacommons.org/licenses/odbl/1.0/). The Open Database License (ODbL) is a license agreement intended to allow users to freely share, modify, and use this Dataset while maintaining this same freedom for others, provided that the original source and author(s) are credited.Mingzhu Dou

Supplementary material 5 TLC test using C solvent system Data type jpg Explanation note a: fresh plate under 254 nm ultraviolet light; b: charring plate under daylight; c: charring plate under 365 nm ultraviolet light. 0: standard substance (*Lethariellacladonioides*, norstictic acid at Rf four and atranorin at Rf seven). a: 1: ZJ240446 (*Malmidea* sp.); 2: ZJ240088 (*Allographa* sp.); 3: ZJ240057 (*Phyllobaeis* sp.); 4: ZJ240058 (*Phyllobaeis* sp.); 5: ZJ240059 (*Phyllobaeis* sp.); 6: ZJ240060 (*Pyrrhospora* sp.); 7: ZJ240084 (*Allographa* sp.); 8: ZJ240097 (*Pyrrhospora.* sp.); 9: ZJ240098 (*Pyrrhospora* sp.); 10: ZJ240205 (*Pyrrhospora.* sp.); 11: ZJ240253 (*Allographa* sp.); 12: ZJ240318 (*Allographa* sp.); 13. ZJ240323 (*Allographa* sp.); 14. ZJ240478 (*Phyrrospora* sp.); 15: HN19676 (*Pyrenula* sp.); 13: HN19701 (*Pyrenula* sp.). https://binary.pensoft.net/file/1173214This dataset is made available under the Open Database License (http://opendatacommons.org/licenses/odbl/1.0/). The Open Database License (ODbL) is a license agreement intended to allow users to freely share, modify, and use this Dataset while maintaining this same freedom for others, provided that the original source and author(s) are credited.Mingzhu Dou

Supplementary material 6 TLC test using C solvent system, charring plate under 365 nm ultraviolet light Data type jpg Explanation note 1: ZJ240681 (*Allographa* sp.); 2: ZJ240697 (*Graphis* sp.); 3: ZJ240679 (*Graphis* sp.); 4: ZJ240680 (*Graphis* sp.); 5: ZJ240691 (*Graphis* sp.); 6: ZJ240694 (*Graphis* sp.); 7: ZJ240528 (*Coenogonium* sp.); 8: ZJ232073 (*Thelotrema* sp.); 9: ZJ240421 (*Ocellularia* sp.); 10: ZJ240226 (*Platythecium* sp.); 11: ZJ240334 (*Coenogonium* sp.); 12: ZJ240325 (*Coenogonium* sp.); 13. FJ231826 (*Phyllobaeis* sp.); 14. HNX241177 (*Coenogonium* sp.). https://binary.pensoft.net/file/1173215This dataset is made available under the Open Database License (http://opendatacommons.org/licenses/odbl/1.0/). The Open Database License (ODbL) is a license agreement intended to allow users to freely share, modify, and use this Dataset while maintaining this same freedom for others, provided that the original source and author(s) are credited.Mingzhu Dou
