# Supplementary material for: ﻿Phylogenetic analysis shows that Pyrenula (Pyrenulaceae) diversity is larger than expected: three new species and one new record discovered in China
Source: MycoKeys. 2024 Nov 13;110:159–83. doi: 10.3897/mycokeys.110.131741 (PMC11579651; doi:10.3897/mycokeys.110.131741)
Supplement: Supplementary material 1 — Information for the sequences download from NCBI used in this study [file mycokeys-110-159-s001.docx]

Information for the sequences download from NCBI used in this study.

| **Species Name** | **Specimen No.** | **Locality** | **GenBank accession number** | | |
| --- | --- | --- | --- | --- | --- |
|  |  |  | **ITS** | **nuLSU** | **mtSSU** |
| *Pyrenula thailandicoides* M.Z. Dou & Z.F. Jia | FJ220208 | China Fujian | OR578593 | — |  |
| *P. thailandicoides* M.Z. Dou & Z.F. Jia | YN18212 | China Yunnan | OR578589 | OR578570 |  |
| *P. thailandicoides* M.Z. Dou & Z.F. Jia | YN18015 | China Yunnan | OR578590 | OR578571 |  |
| *P. inspersa* M.Z. Dou & Z.F. Jia | HN17058 | China Hainan | OR578591 | OR578572 |  |
| *P. apiculata* M.Z. Dou & Z.F. Jia | YN18172 | China Yunnan | OR578592 | OR578573 |  |
| *P.* cf. *acutalis* R.C. Harris | F_19092_b | Australia | — | DQ329026 | DQ329001 |
| *P.* aff. *aggregataspistea* Aptroot & M. Cáceres | AA11618 | Brazil | — | KT808561 | — |
| *P. aggregataspistea* Aptroot & M. Cáceres | AA11216 | Brazil | KT820112 | KT808557 | KT808487 |
| *P. anomala* (Ach.) A. Massal. | AA11222 | Brazil | KT820168 | KT808607 | KT808544 |
| *P. anomala* (Ach.) A. Massal. | AA11607 | Brazil | KT820116 | — | KT808490 |
| *P. anomala* (Ach.) A. Massal. | AA15591 | Brazil | KT820113 | — | KT808486 |
| *P. arthoniotheca* Upreti | AA11887 | Brazil | KT820120 | — | — |
| *P. aspistea* (Ach.) Ach | AA11263 | Brazil | KT820121 | KT808560 | KT808491 |
| *P. aspistea* (Ach.) Ach | AA13547 | Brazil | KT820123 | — | — |
| *P. aspistea* (Ach.) Ach | CBS_109078 | Hong Kong | — | EF411063 | — |
| *P. aspistea* (Ach.) Ach | CG3030 | Vietnam | KT820124 | KT808562 | KT808494 |
| *P. aspistea* (Ach.) Ach | CG3060 | Vietnam | KT820125 | KT808564 | KT808495 |
| *P. aspistea* (Ach.) Ach | CG3070 | Vietnam | KT820126 | — | — |
| *P. aspistea* (Ach.) Ach | CG3071 | Vietnam | KT820127 | — | — |
| *P. aspistea* (Ach.) Ach | GW1042 | Sri Lanka | JQ927450 | JQ927469 | — |
| *P. aspistea* (Ach.) Ach | GW1044 | Sri Lanka | JQ927451 | JQ927470 | JQ927462 |
| *P. aspistea* (Ach.) Ach | RAMK17271 | Thailand | KT820128 | — | KT808492 |
| *P. aspistea* (Ach.) Ach | RAMK17277 | Thailand | KT820129 | KT808563 | KT808493 |
| *P. astroidea* (Fée) R.C. Harris | RAMK17281 | Thailand | KT820088 | — | — |
| *P. astroidea* (Fée) R.C. Harris | RVH7 | Laos | KT820089 | KT808565 | KT808496 |
| *P. bahiana* Malme | RVH1 | Laos | KT820090 | — | — |
| *P. bahiana* Malme | RVH2 | Laos | KT820091 | KT808614 | — |
| *P. bahiana* Malme | RVH3 | Laos | KT820092 | KT808605 | KT808498 |
| *P. balia* (Kremp.) R.C. Harris | CG3063 | Vietnam | KT820130 | KT808566 | KT808499 |
| *P. brunnea* Fée | CG3023 | Vietnam | KT820093 | — | — |
| *P.* cf*. subglabrata* (Nyl.) Müll. Arg | CG3028 | Vietnam | KT820140 | KT808574 | KT808509 |
| *P. chlorospila* (Nyl.) Arnol | CG1520b | England | JQ927452 | JQ927471 | JQ927463 |
| **Species Name** | **Specimen No.** | **Locality** | **GenBank accession number** | | |
|  |  |  | **ITS** | **nuLSU** | **mtSSU** |
| *P. cornutispora* Aptroot & M. Cáceres | AA11938 | Brazil | KT820131 | KT808618 | KT808500 |
| *P. cornutispora* Aptroot & M. Cáceres | ISE_AA11938 | Brazil | NR_158911 | NG_060160 | — |
| *P. corticata* (Müll. Arg.) R.C. Harris | AA11443 | Brazil | KT820132 | KT808568 | KT808501 |
| *P. corticata* (Müll. Arg.) R.C. Harris | AA11466 | Brazil | KT820133 | KT808569 | KT808502 |
| *P. confinis* (Nyl.) R.C. Harris | AA13575 | Brazil | — | KT808567 | KT808550 |
| *P. cruenta* (Mont.) Vain | Green_PYCR12 | USA | KC592268 | — | — |
| *P. cruenta* (Mont.) Vain | Green_PYCR16 | USA | KC592269 | — | — |
| *P. cruenta* (Mont.) Vain | Green_PYCR4 | USA | KC592267 | — | — |
| *P. cruenta* (Mont.) Vain | Lutzoni_9806174 | Puerto Rico | — | AF279407 | — |
| *P. fetivica* (Kremp.) Müll. Arg | CG1963 | Vietnam | KT820134 | — | KT808503 |
| *P. fetivica* (Kremp.) Müll. Arg | GW307A | Sri Lanka | JQ927453 | JQ927472 | JQ927464 |
| *P. fetivica* (Kremp.) Müll. Arg | GW835 | Sri Lanka | JQ927454 | — | JQ927465 |
| *P. infraleucotrypa* Aptroot & M. Cáceres | AA11105 | Brazil | KT820114 | KT808558 | KT808489 |
| *P. infraleucotrypa* Aptroot & M. Cáceres | AA11468 | Brazi | KT820136 | — | — |
| *P. infraleucotrypa* Aptroot & M. Cáceres | AA11499 | Brazi | KT820115 | — | — |
| *P. infraleucotrypa* Aptroot & M. Cáceres | AA15450 | Brazi | KT820142 | KT808575 | KT808510 |
| *P. infraleucotrypa* Aptroot & M. Cáceres | AA15451 | Brazi | KT820117 | KT808559 | KT808488 |
| *P. inframamillana* Aptroot & M. Cáceres | AA11220 | Brazi | KT820137 | KT808572 | KT808506 |
| *P. inframamillana* Aptroot & M. Cáceres | AA11272 | Brazi | KT820138 | KT808571 | KT808507 |
| *P. inframamillana* Aptroot & M. Cáceres | AA11897 | Brazi | KT820139 | KT808573 | KT808508 |
| *P. laevigata* (Pers.) Arnold | OL_206758 | Norway | MK812685 | — | — |
| *P. laevigata* (Pers.) Arnold | OL_206773 | Norway | MK812185 | — | — |
| *P. laevigata* (Pers.) Arnold | Palice 5608 | Slovakia | — | AY607736 | AY568029 |
| *P.* cf*. leucostoma* Ach. | F_19082 | Australia | — | DQ329024 | DQ328999 |
| *P. macrospora* (Degel.) Coppins & P. James | CG1520a | England | JQ927455 | JQ927473 | JQ927466 |
| **Species Name** | **Specimen No.** | **Locality** | **GenBank accession number** | | |
|  |  |  | **ITS** | **nuLSU** | **mtSSU** |
| *P. mamillana* (Ach.) Trevis. | AA11342 | Brazil | KT820143 | KT808576 | KT808515 |
| *P. mamillana* (Ach.) Trevis. | AA11610 | Brazil | KT820144 | KT808615 | KT808516 |
| *P. mamillana* (Ach.) Trevis. | AA11846 | Brazil | KT820145 | KT808617 | KT808517 |
| *P. mamillana* (Ach.) Trevis. | AA15465 | Brazil | KT820146 | KT808579 | KT808519 |
| *P. mamillana* (Ach.) Trevis. | CG3014 | Vietnam | KT820147 | KT808580 | KT808511 |
| *P. mamillana* (Ach.) Trevis. | CG3034 | Vietnam | KT820149 | KT808582 | KT808514 |
| *P. mamillana* (Ach.) Trevis. | CG3058 | Vietnam | KT820150 | KT808583 | KT808518 |
| *P. mamillana* (Ach.) Trevis. | CG3059 | Vietnam | KT820151 | KT808584 | KT808513 |
| *P.* aff. *mamillana* (Ach.) Trevis. | GW818A | Sri Lank | JQ927456 | JQ927474 | JQ927467 |
| *P. massariospora* (Starbäck) R.C. Harris | CG3061 | Vietnam | KT820153 | KT808585 | KT808521 |
| *P. massariospora* (Starbäck) R.C. Harris | CG3062 | Vietnam | KT820154 | KT808586 | KT808522 |
| *P. massariospora* (Starbäck) R.C. Harris | GW1028 | Sri Lanka | JQ927457 | JQ927475 | JQ927468 |
| *P. minor* Fée | AA11505 | Brazil | KT820155 | KT808620 | KT808524 |
| *P. minor* Fée | AA13516 | Brazil | — | KT808587 | KT808523 |
| *P. minutispora* Aptroot & M. Cáceres | AA11877 | Brazil | KT820119 | — | — |
| *P. minutispora* Aptroot & M. Cáceres | ABL_AA11877 | Brazil | NR_136140 | — | — |
| *P. nitida* (Weigel) Ach. | 17076 | Poland | MN387114 | — | — |
| *P. nitida* (Weigel) Ach. | 17081 | Poland | MN387115 | — | — |
| *P. nitida* (Weigel) Ach. | 17146 | Poland | MN387116 | — | — |
| *P. nitida* (Weigel) Ach. | 17189 | Poland | MN387117 | — | — |
| *P. nitida* (Weigel) Ach. | F_5929 | Czech Republic | JQ927458 | DQ329023 | DQ328998 |
| *P. nitida* (Weigel) Ach. | s. n. | Germany | — | AY607737 | AY568030 |
| *P. nitidella* (Flörke) Müll. Arg. | 17082 | Poland | MN387139 | — | — |
| *P. nitidella* (Flörke) Müll. Arg. | CG3027 | Vietnam | KT820156 | — | KT808525 |
| *P. occidentalis* (R.C. Harris) R.C. Harris | OL_206777 | Norway | MK811633 | — | — |
| *P. ochraceoflava* (Nyl.) R.C. Harris | Gaya_160308_EGB11 | USA | KC592275 | — | KC592289 |
| *P. paraminarum* Aptroot & M. Cáceres | AA11012 | Brazil | KT820135 | KT808570 | KT808504 |
| *P. paraminarum* Aptroot & M. Cáceres | AA11471 | Brazil | — | — | KT808526 |
| *P. punctella* (Nyl.) Trevis*.* | Tripp4522 | — | KT232213 | — | KT276274 |
| **Species Name** | **Specimen No.** | **Locality** | **GenBank accession number** | | |
|  |  |  | **ITS** | **nuLSU** | **mtSSU** |
| *P. pyrenuloides* (Mont.) R.C. Harris | CG1545 | Vietnam | KT820094 | — | — |
| *P. quassiicola* Fée | CG3001 | Vietnam | KT820098 | KT808588 | KT808528 |
| *P. quassiicola* Fée | CG3019 | Vietnam | KT820101 | KT808591 | KT808531 |
| *P. quassiicola* Fée | CG3032 | Vietnam | KT820104 | KT808592 | — |
| *P. quassiicola* Fée | CG3033 | Vietnam | KT820105 | KT808593 | — |
| *P. quassiicola* Fée | RVH6 | Laos | KT820107 | KT808595 | KT808535 |
| *P. sanguinea* Aptroot, M. Cáceres & Lücking | 15707F | Brazil | — | KF697129 | KF697128 |
| *P. leucostoma* Aptroot & Gueidan | AFTOL_ID387 | USA | DQ782845 | — | — |
| *P. leucostoma* Aptroot & Gueidan | DUKE_0047599 | — | NR_119610 | NG_068722 | — |
| *P. leucostoma* Aptroot & Gueidan | Reeb VR 14 VI 025 | USA | — | AY640962 | — |
| *P. reginae* E.L. Lima, Aptroot & M. Cáceres | ELL0010 | Brazil | — | KT808596 | — |
| *P. rubronitidula* Aptroot & M. Cáceres | AA11332 | Brazil | KT820157 | KT808597 | — |
| *P. rubronitidula* Aptroot & M. Cáceres | AA15603 | Brazil | KT820158 | — | — |
| *P. rubrostigma* Aptroot & M. Cáceres | AA11697 | Brazil | KT820159 | KT808616 | KT808539 |
| *P. rubrostigma* Aptroot & M. Cáceres | ISE_AA11697 | Brazil | NR_158913 | NG_06015 | — |
| *P. scutata* (Stirt.) Zahlbr | CG1635 | Vietnam | KT820160 | KT808598 | KT808540 |
| *P. septicollaris* (Eschw.) R.C. Harris | AA13534 | Brazil | KT820166 | KT808610 | KT808551 |
| *P. septicollaris* (Eschw.) R.C. Harris | AA13546 | Brazil | KT820161 | — | — |
| *P. septicollaris* (Eschw.) R.C. Harris | AA13555 | Brazil | KT820167 | — | — |
| *P. septicollaris* (Eschw.) R.C. Harris | AA15009 | Brazil | — | KT808599 | KT808541 |
| *P. septicollaris* (Eschw.) R.C. Harris | AA15012 | Brazil | KT820162 | KT808600 | — |
| *P. septicollaris* (Eschw.) R.C. Harris | AA15021 | Brazil | KT820163 | KT808601 | KT808542 |
| *P. septicollaris* (Eschw.) R.C. Harris | AA15023 | Brazil | KT820164 | KT808602 | — |
| *P. septicollaris* (Eschw.) R.C. Harris | AA15038 | Brazil | — | KT808603 | — |
| *P. septicollaris* (Eschw.) R.C. Harris | AA15042 | Brazil | KT820165 | KT808604 | — |
| *P. sexlocularis* (Eschw.) R.C. Harris | RAMK17261 | Thailand | KT820108 | KT808606 | KT808543 |
| *P.* sp. | F19113n | Australia | — | DQ329027 | DQ329002 |
| *P.* sp. | CG3009 | Vietnam | KT820110 | KT808611 | KT808547 |
| *P.* sp*.* | F19082r | Australia | JQ927461 | DQ329025 | DQ329000 |
| *P.* sp. | LHD210 | Vietnam | AB935436 | — | — |
| **Species Name** | **Specimen No.** | **Locality** | **GenBank accession number** | | |
|  |  |  | **ITS** | **nuLSU** | **mtSSU** |
| *P. subelliptica* (Tuck.) R.C. Harris | RVH5 | Laos | KT820106 | KT808594 | KT808534 |
| *P. subglabrata* (Nyl.) Müll. Arg. | CG3069 | Vietnam | KT820169 | KT808608 | KT808545 |
| *P. subpraelucida* Müll. Arg. | F_17550_f | Costa Rica | — | DQ329015 | DQ328986 |
| *P. thelomorpha* Tuck. | CG3056 | Australia | JQ927460 | KT808609 | KT808546 |
| *P. viridipyrgilla* Aptroot & M. Cáceres | AA11864 | Brazil | KT820170 | KT808619 | KT808548 |
| *P. viridipyrgilla* Aptroot & M. Cáceres | ISE_AA11864 | Brazil | NR_158914 | — | — |
| *Cyphellophora europaea* (de Hoog, Mayser & Haase) Réblová & Unter. | CBS129_96 | — | EF551553 | FJ358248 | FJ225750 |
| *Endocarpon pusillum* Hedw. | CG470 | — | JQ927447 | EF643754 | FJ225677 |
