# Supplementary figures and images for: ﻿Phylogenetic analysis shows that Pyrenula (Pyrenulaceae) diversity is larger than expected: three new species and one new record discovered in China
Source: MycoKeys. 2024 Nov 13;110:159–83. doi: 10.3897/mycokeys.110.131741 (PMC11579651; doi:10.3897/mycokeys.110.131741)

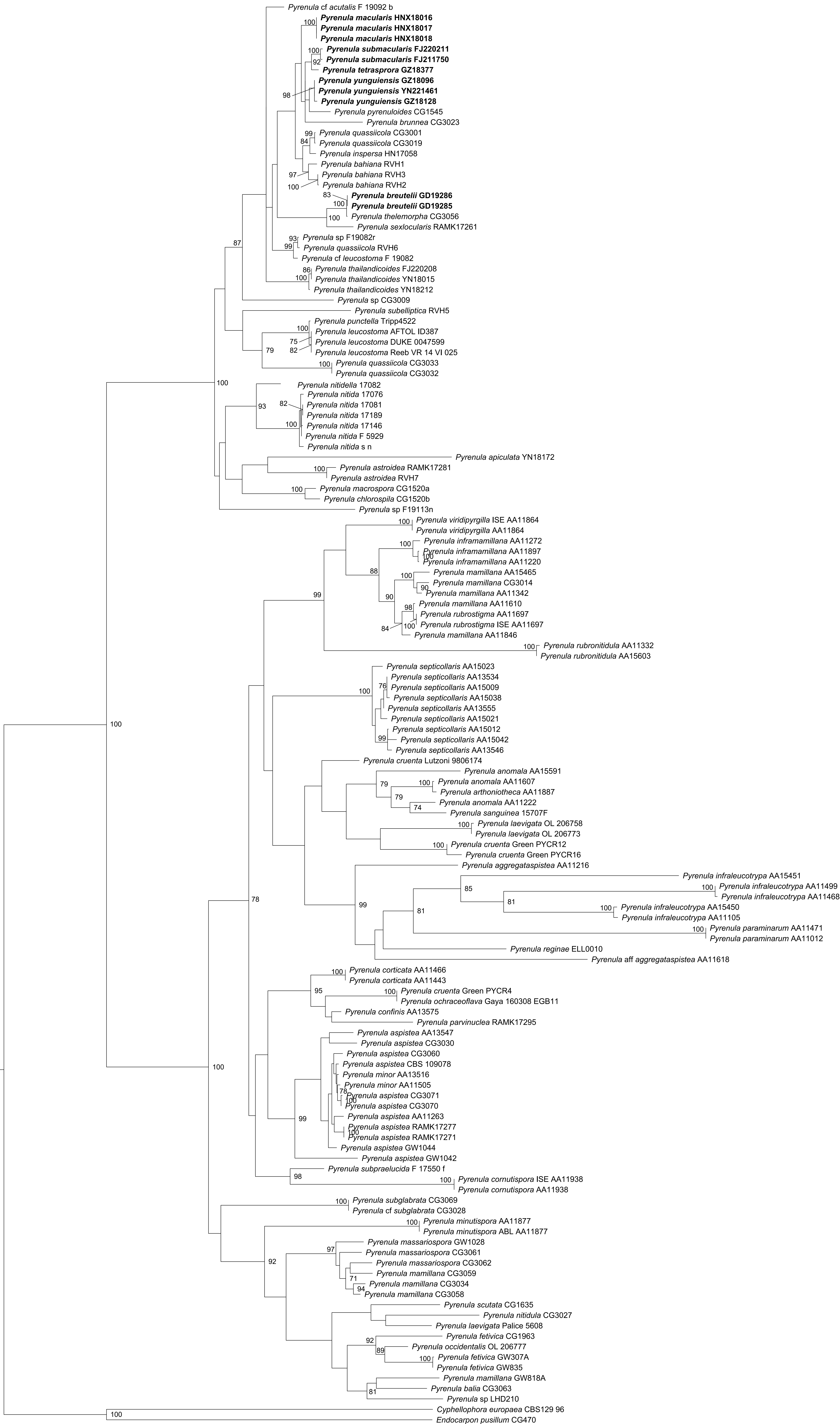

Supplement: Supplementary material 2 — Phylogenetic tree constructed through ML analyses based on mtSSU, ITS, and nuLSU for Pyrenula [file mycokeys-110-159-s002.pdf]

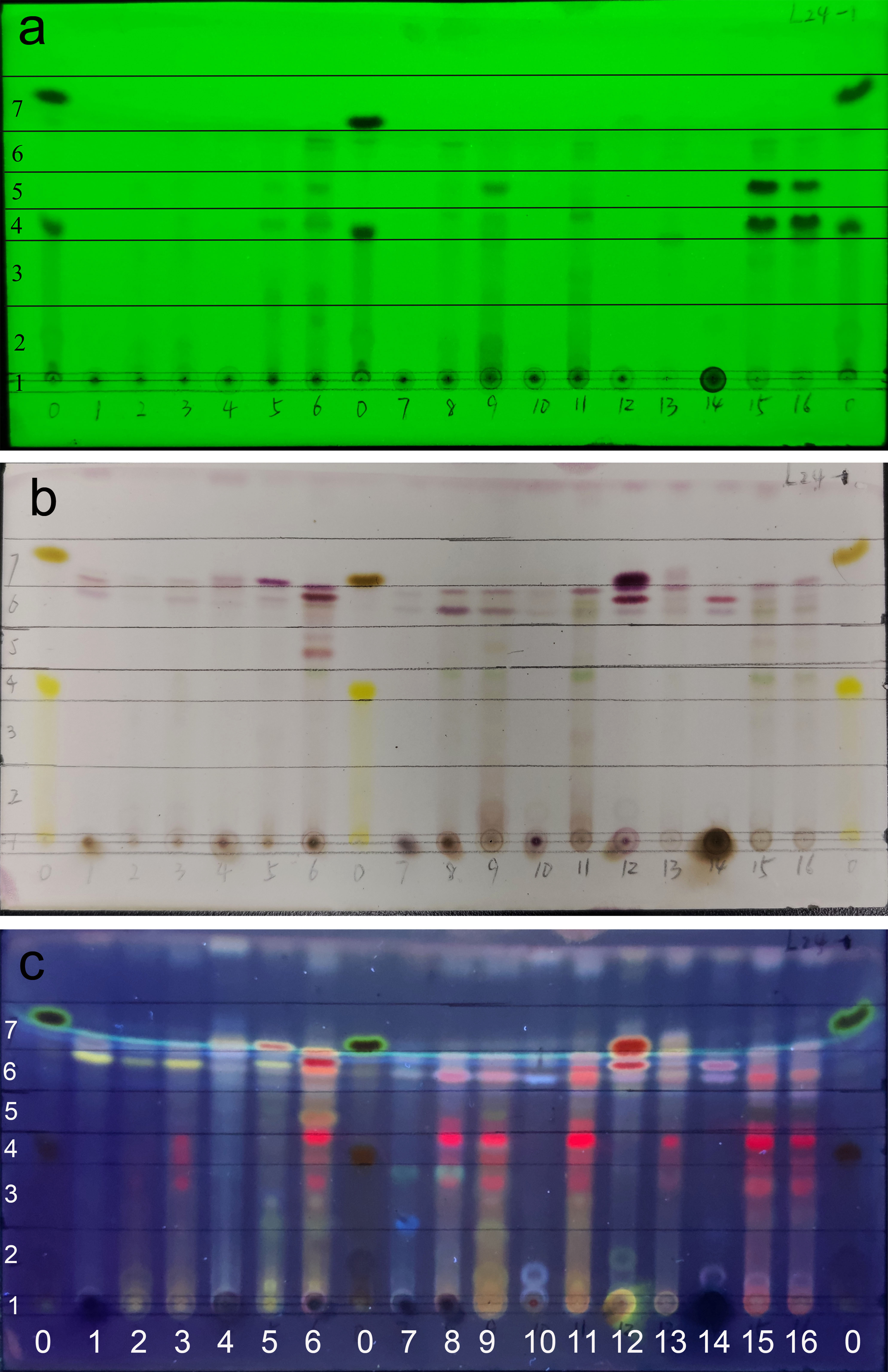

Supplement: Supplementary material 3 — TLC test using C solvent system [file mycokeys-110-159-s003.jpg]

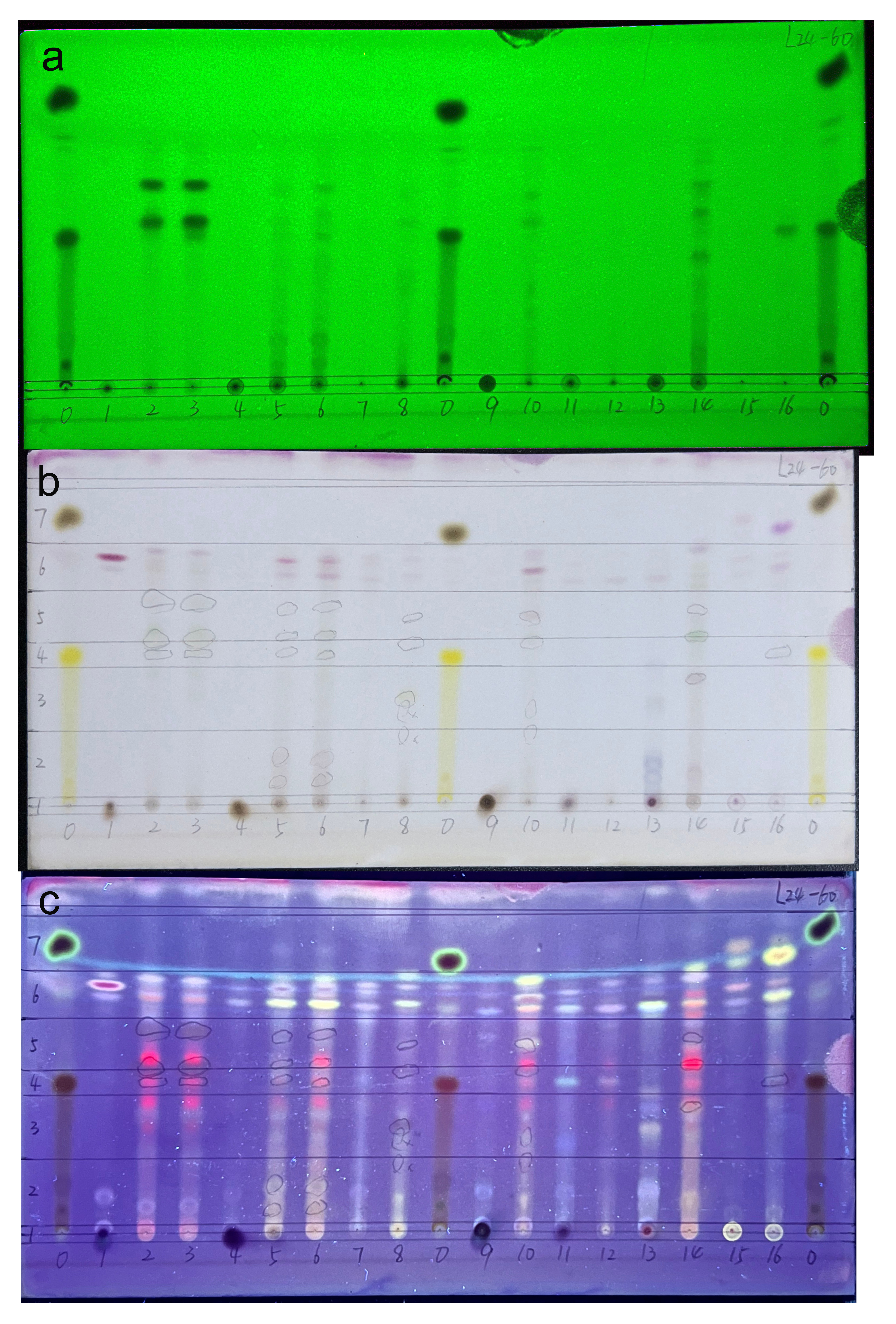

Supplement: Supplementary material 4 — TLC test using C solvent system [file mycokeys-110-159-s004.jpg]
